# MicroRNA-15b Modulates Molecular Mediators of Blood Induced Arthropathy in Hemophilia Mice

**DOI:** 10.3390/ijms17040492

**Published:** 2016-04-08

**Authors:** Dwaipayan Sen, Giridhara R. Jayandharan

**Affiliations:** 1 Department of Hematology, Christian Medical College, 632004 Vellore, India; dsen@cmcvellore.ac.in; 2 Department of Biological Sciences and Bioengineering, Indian Institute of Technology, 208016 Kanpur, India

**Keywords:** microRNA, factor VIII, hemophilia A, murine model

## Abstract

The development of arthropathy is a major co-morbidity in patients with hemophilia. The present study was designed to study the role of a microRNA biomarker (miR-15b) in the development of joint disease. To investigate the expression profile of miR-15b during the development of arthropathy, we first isolated and studied small RNA from the acute and chronic hemarthrosis model of hemophilia A mice. We observed that miR-15b was consistently repressed (~1- to 4-fold) from the onset of joint bleeding (1, 3, 7 and 24 h) until six bleeding episodes (up to 90 days). To test if reconstitution of miR-15b modulates biomarkers of joint damage in a chronic hemarthrosis model, we administered an adeno-associated virus (AAV) 5-miR-15b vector intra-articularly alone or in combination with systemic administration of AAV2-factor VIII. miR-15b overexpression downregulated markers of angiogenesis and hypoxia (vascular epithelial growth factor α (VEGF-α) and hypoxia inducing factor 2α (HIF-2α), ~70% and ~34%, respectively) in the affected joints. In addition, the co-administration of miR-15b and factor VIII vectors reduced the levels of the chondrodegenerative matrix-metalloproteinases (MMPs) 1, 3, 9 and 14 (~14% to 60%) in the injured joints. These data demonstrate for the first time the role of a miR-15b in the development of hemophilic arthropathy and has implications in development of miR based therapies for joint disease.

## 1. Introduction

Blood-induced joint damage or arthropathy is a major cause of morbidity in patients with hemophilia. The pathobiology of hemophilic arthropathy appears multi-factorial. Accumulation of excess blood and iron in joints is known to be etiological [1,2]. After an acute intra-articular bleed, autolysis of erythrocytes results in the deposition of cytoplasmic iron in the synovium and chondrocytes of articular cartilage [3]. Within four days, neovascularization of the sub-synovium and focal areas of villous formation can be detected on the synovial surface, resulting in synovial hypertrophy, which is friable and more likely to re-bleed with minimal stress [4]. This evolves into a chronic, persistent inflammatory disorder termed “hemophilic synovitis”. A vicious cycle of re-bleeding is then established, creating a target joint. Repeated bleeding into the target joint is associated progressive inflammation of joint capsule. Eventually, these processes lead to complete erosion of the cartilage and development of arthropathy [5,6]. The molecular mediators involved in this process are not well known.

We have recently investigated the molecular signaling events contributing to arthropathy in a murine model of hemophilia [7]. We found that during hemarthrosis, nuclear factor kappa B (NF-κB), a key stress-responder and inflammatory mediator and other molecular markers of hypoxia (hypoxia inducing factor 1α (HIF-1α) and HIF-2α), angiogenesis (vascular epithelial growth factor α (VEGF-α)) and chondro-degenerative matrix-metalloproteinases 3 (MMP3) and MMP13 play a progressive role in the development of arthropathy. Some of these factors have also been implicated in other *in vitro* studies thus confirming their role in progression of joint disease [8,9]. Since these factors have pleotropic cellular functions, we reasoned that identification of small RNAs contributing to joint damage will be beneficial for specific targeting.

Dysregulated expression of microRNA (miR) has been noted in a variety of inflammatory joint diseases [10]. Since miR based biomarkers are not known in hemophilic joint disease, establishing a pattern of miR expression from the onset of bleeding to the development of arthropathy will be informative. In our recent studies [7], we identified that expression of a small RNA, miR-15b, was attenuated (~2.5-fold) during a single episodic bleeding, 3 h after initial bleeding. Interestingly, miR-15b is known to play a critical role in apoptosis by targeting B-cell lymphoma 2 (BCL2) [11,12,13] and negatively regulates proangiogeneic factors like VEGF-α [14,15] as well as hypoxia regulating factors [14]. Because many of these are part of the pathogenesis in hemophilic arthropathy [7], we reasoned that targeted overexpression of miR-15b may modulate molecular mediators of arthropathy. In addition, we also investigated the effect of miR-15b on various MMPs (1–17), as these chondro-degenerative enzymes are the crucial mediators of cartilage turnover and articular damage [16].

## 2. Results

### 2.1. miR-15b Expression Is Altered during the Development of Arthropathy in Vivo

To document the expression pattern of miR-15b during development of arthropathy, the acute hemarthrosis model was studied 1, 3, 7 and 24 h after a single-injury and the chronic hemarthrosis model was studied at 60, 75 and 90 days for up to six injuries. These representative time-points were selected to profile the trend of miR-15b levels in the injured joint tissue immediately after a single bleed or after multiple bleeds. These models recapitulated the gross and histomorphologic changes associated with synovitis and hemophilic arthropathy as previously described [7]. For these studies, a control of hemostatically normal mice was not included, as our objective was to profile relative miR-15b levels between injured and control joints in hemophilic mice. Nonetheless, we and others have shown that the joint inflammation scores or the levels of inflammatory cytokines in synovial fluid of after an single or multiple injury is not altered in hemostatically normal mice [7,17].

**Figure 1:**
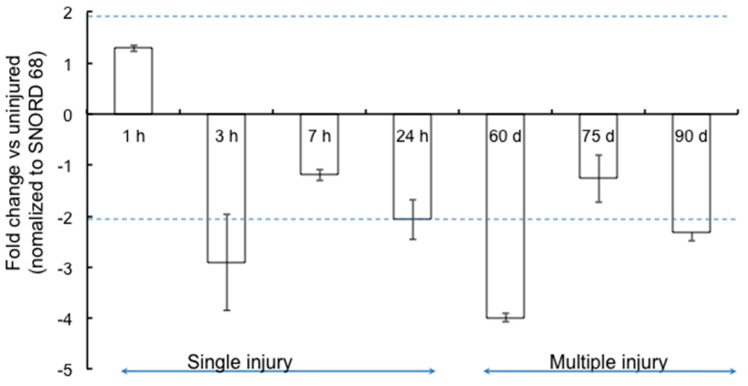
Expression profile of miR-15b in the joints following single (1, 3, 7 and 24 h) or multiple (4–6) bleeds (Days 60, 75 and 90). The levels of miR-15b were assessed in joint RNA by qPCR using predesigned locked nucleic acid (LNA) primers as described in the “Materials and Methods”. Small Nucleolar RNA, C/D Box 68 (SNORD68) levels were used as the housekeeping control for normalization. Data is representative of mean ± SD of three replicates. *p* < 0.05 is considered to be statistically significant.

Joint specific RNA was isolated at each of these time-points and their specificity confirmed by amplification of chondrocyte-specific collagen type 2 A (COL2A) gene. We then assayed miR-15b expression by qPCR at each one of these time points. As seen in Figure 1, miR-15b was downregulated (~1- to 4-fold) from 3 h after a single bleeding episode to the 90-day time point where the animals have two to six bleeds into the joint cavity. The maximal down regulation (~4-fold) of miR-15b was observed at 60 days. These data suggest that miR-15b is consistently repressed from the onset of synovitis to the development of arthropathy in the hemarthrosis model.

### 2.2. miR-15b Vectors Are Functional in Vitro

**Figure 2:**
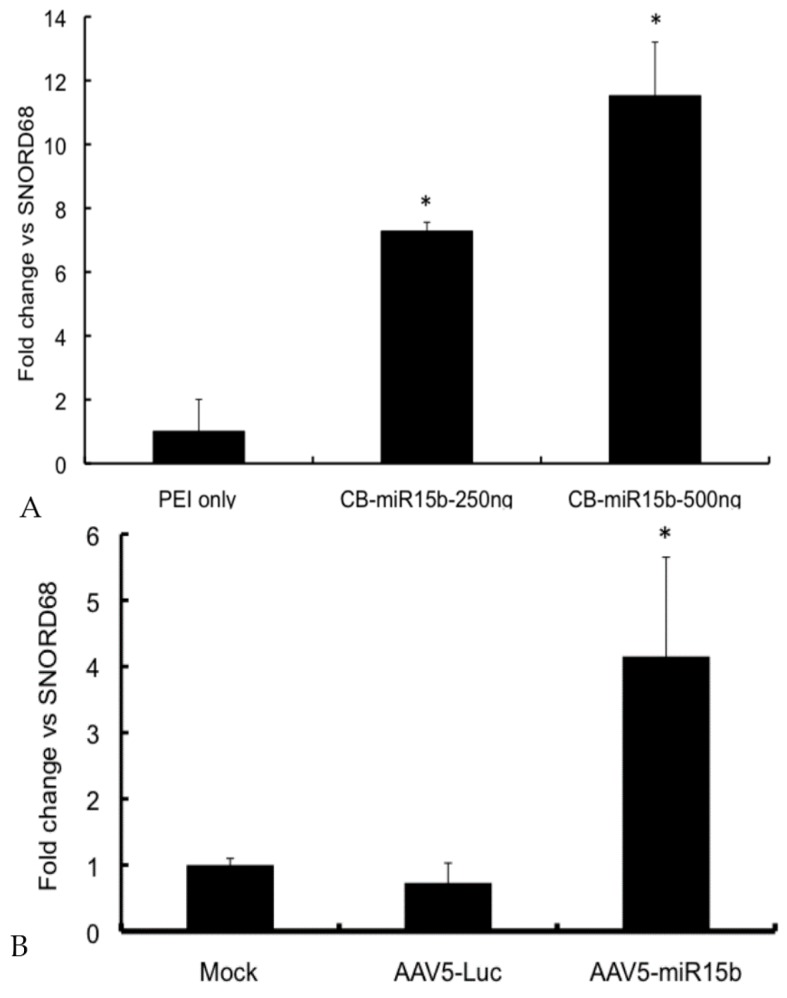
Validation of the miR-15b vectors *in vitro*: (**A**) *In vitro* expression of miR-15b in NIH3T3 cells. About 250 ng and 500 ng CB-miR-15b plasmid was used to transfect NIH3T3 cells in a 24 well plate using Polyethylenimine (PEI) as the transfection agent (1 mg/mL). 48 h post transfection, total RNA was harvested and qPCR performed to detect mature miR-15b. The data shown are mean ± SD of two independent experiments; (**B**) Expression of miR-15b in HeLa cells following infection with adeno-associated virus (AAV) 5-miR-15b vectors. NIH-3T3 cells were mock infected or infected with 5 × 10^3^ vgs/cell of either AAV5-Luc or AAV5-miR-15b vectors. 48 h post infection, cells were harvested and total RNA was isolated to measure miR-15b levels. Data shown are mean ± SD of two independent experiments done in triplicates and normalized to mock control group. * *p* < 0.05 when compared to the controls.

To determine if overexpression of CB-miR-15b plasmid results in any toxicity to the recipient cells, a dose-finding study was performed with an MTT assay. As shown in Supplementary Material Figure S1, there was no significant toxicity of the plasmids at the dose range of 50–500 ng. We then evaluated if the new construct is functional and is able to express the encoded miR. NIH3T3 cells were transfected with 250 and 500 ng of the plasmid. Forty-eight hours later, total RNA was isolated and the levels of miR-15b were determined by qPCR with SNORD68 as the housekeeping control gene. As seen in Figure 2A, there was a dose-dependent increase in miR-15b expression (~7–11-fold) when compared to the control samples. Once validated, the plasmids were used to package adeno-associated virus (AAV) 5-miR-15b vectors, which expressed high levels (~5.7-fold) of the transgene, miR-15b (Figure 2B). Data from these studies confirm that the miR-15b vectors are functional and express mature miR-15b.

### 2.3. Kinetics of Adeno-Associated Virus (AAV) Mediated Transgene Expression in Joint Tissue of Hemarthrosis Model

**Figure 3:**
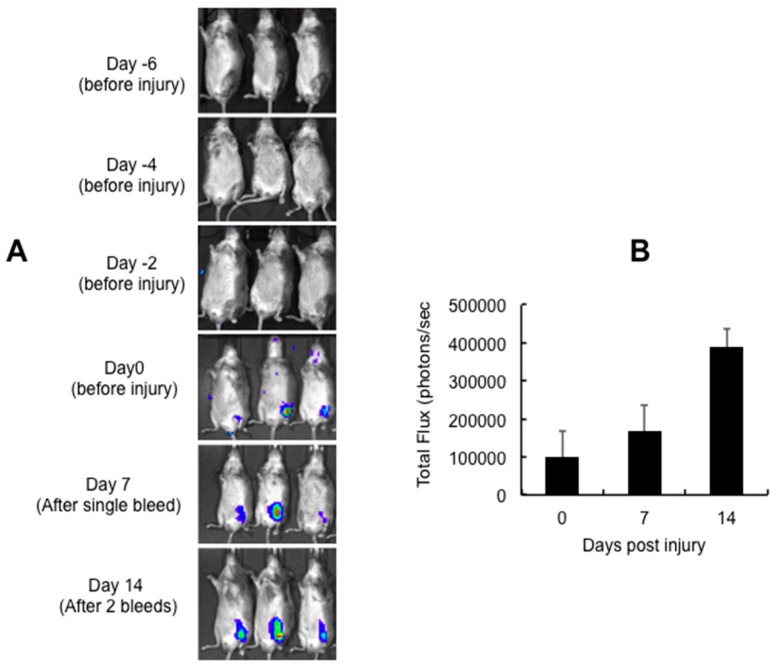
Persistence of intra-articular gene (luciferase) expression following multiple episodic bleeding. Approximately 5 × 10^9^ vector particles (in a volume of 10 μL) of AAV5-Luc was injected into the left knee of 10-week-old C57BL/6 mice, seven days before the first injury (Day 0): (**A**) intra-articular luciferase expression at different days post vector administration; and (**B**) quantitative data of transgene expression 0, 7 and 14 days post injury. Images for bioluminescence were captured in an IVIS Spect-CT small animal imaging system (Perkin Elmer, Caliper Life Sciences, Hopkinton, MA, USA).

AAV5 is known to be an efficient vector for intra-articular gene delivery when compared to AAV2 and AAV8 vectors [17,18,19,20]. We thus selected AAV5 serotype to express miR-15b into the joints of the hemarthrosis model. In our first set of *in vivo* studies, we wished to determine the time frame for transgene expression from an AAV5 vector after their administration into the joint tissue. We thus administered ~5 × 10^9^ vector genomes (vgs) of AAV5-luc in knee joints of hemophilia A mice. Our results demonstrate that luciferase expression is first detected on Day 7 after the intra-articular injection of AAV5-Luc vectors (Figure 3A). Subsequently, we tested if these vectors persist in the joints after multiple bleedings/injuries. As can be seen in Figure 3, luciferase expression was consistently detected in the knee joint even after two bleeding episodes. This indicates that the articular damage caused by multiple injuries does not affect the persistence of AAV5 vector in joint tissue. Interestingly, AAV5 has been shown to transduce both human fibroblast like synovial cells and chondrocytes or murine joints very efficiently [17,18,19,20].

**Figure 4:**
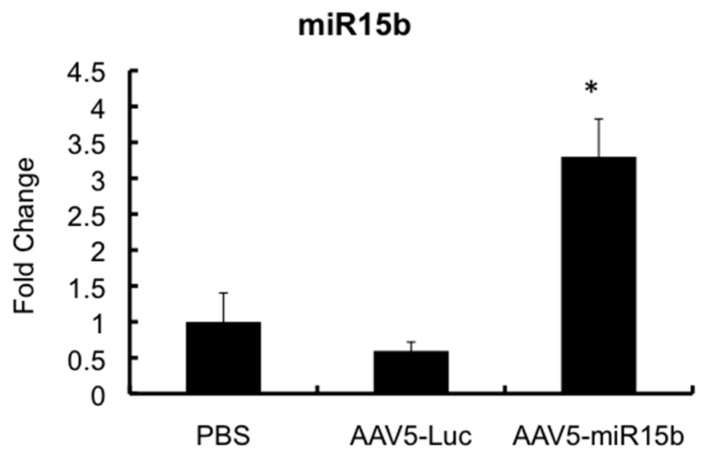
Validation of miR-15b levels in the joints of hemophilia A mice following multiple injuries. Approximately 5 × 10^9^ vector particles (in a volume of 10 μL) of AAV5-miR-15b was injected into the left knee of 10-week-old mice, seven days before the first injury (Day 0). After two injuries, joint specific RNA was isolated and miR-15b levels were determined by qPCR. SNORD68 was used as the housekeeping gene for normalization. * *p* < 0.05 when compared to the control joints administered with PBS or AAV-Luc vectors.

We then evaluated if the AAV5 vector expressing the miR-15b transgene is functional *in vivo*. To test this, hemophilia A mice were administered with 5 × 10^9^ vgs of AAV5-miR-15b vectors and two injuries were performed seven days apart. Mice that received equal dose of an AAV5-Luc vector served as the control group. After 21 days of intra-articular vector injection and two bleeding episodes, joint specific RNA was isolated and miR-15b transcripts were measured by qPCR. As seen in Figure 4, there was a significant increase (~5.3-fold) in mature miR-15b levels in the joint tissue as compared to the AAV5-Luc injected joints. These data show that miR-15b expression is elevated and persistent in the murine joints even after two bleeding episodes.

### 2.4. Intra-Articular Administration of AAV-miR-15b Attenuates Molecular Mediators of Arthropathy

We had previously established that in the multiple injury model of hemarthrosis (Day 30/60/75/90), factors that contribute to hypoxia (HIF-1α, 3.3–6.5-fold), angiogenesis (VEGF-α, 2.5–4.4-fold) and chondrocyte damage (matrix metalloproteinase 13 (MMP13), 2.8–3.8-fold) were significantly elevated [7]. Further histopathological correlations at equivalent time-points had suggested that these molecular mediators affect the joint scores and lead to arthropathy by Day 60. In the present studies, we observed that miR-15b is maximally repressed at Day 60 (Figure 1). Taken together, the 60-day time point representing the multiple hemarthrosis model was chosen to study the effect of miR-15b over-expression *in vivo*. Groups of hemophilia A mice were thus mock injected or administered with an AAV5-miR-15b vector intra-articularly and/or AAV2-TM-h.FVIII vector administered intravenously. At Day 60 after the first injury (equivalent to Day 67 after gene transfer), we profiled the molecular mediators in the joint tissue.

**Figure 5:**
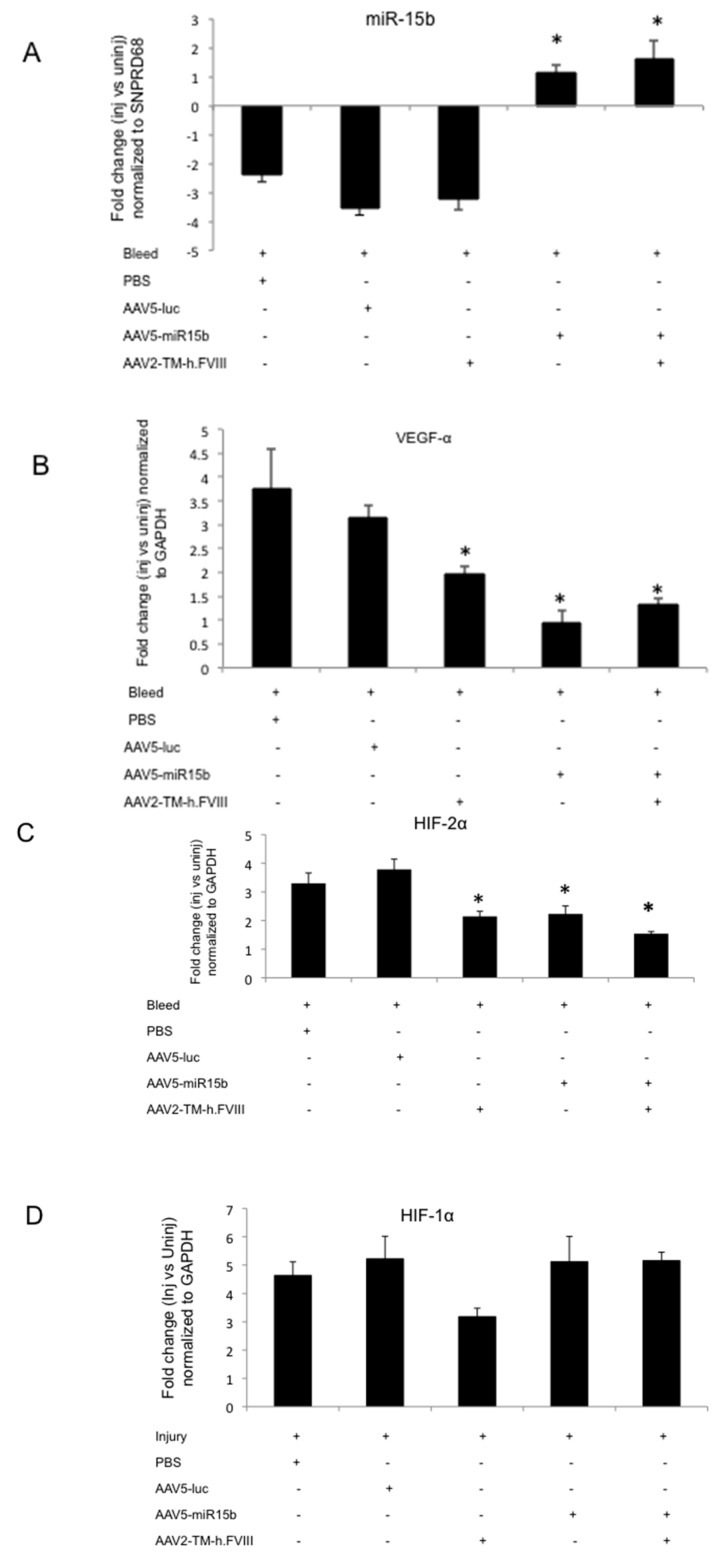
miR-15b over-expression in the murine knee joints after four episodic bleeds downregulates vascular epithelial growth factor α (VEGF-α) and hypoxia inducing factor 2α (HIF-2α) levels but not HIF-1α in the injured joints. Groups of mice (*n* = 4 to 5) were administered with either PBS or AAV5-miR-15b or AAV5-luc vectors (intra-articular) and/or AAV2-TM-h.FVIII (intravenous) vectors. After four episodic bleedings in the left knee joints, qPCR was performed on joint RNA for various targets: (**A**) miR-15b; (**B**) HIF-1α; (**C**) VEGF-α; and (**D**) HIF-2α. * *p* < 0.05 when compared to the AAV5-Luc treated injured animals. GAPDH: Glyceraldehyde 3-phosphate dehydrogenase.

The presence of circulating h.FVIII in animals that received intravenous AAV2-TM-h.FVIII vector was detected by immunoblotting for the light chain of h.FVIII (Supplementary Material Figure S2). At Day 60 post-injury, miR-15b was significantly down-regulated in the injured joints (~3.5-fold) compared to the uninjured control (Figure 5A) which corroborated the data from Figure 1. Conversely, the groups, which received miR-15b or h.FVIII vectors, showed increased expression of miR-15b (~3.4–3.9-fold) in comparison to the control group. These data further validate the persistence and expression of AAV5 vector in the joints, even after four episodic bleedings.

Furthermore, overexpression of miR-15b significantly decreased the levels of proangiogenic VEGF-α (~70%, Figure 5B) and HIF-2α (~34%, Figure 5C) in injured joints when compared to the AAV5-Luc administered control group. The presence of circulating h.FVIII in combination with miR-15b demonstrated a marginal increase in miR-15b (~43%) and VEGF-α (37%) levels while a marginal reduction of HIF-2α (~34%) levels was noted when compared to animals that received miR-15b vectors alone (Figure 5A–C). These changes were statistically insignificant and did not adversely affect the final outcome, of overall reduction in levels of these molecular mediators in comparison to the control (AAV5-Luc treated/injured) animals. There was also no change in the levels of HIF-1α at various conditions tested (Figure 5D). Nonetheless, a combination gene transfer of h.FVIII and miR-15b was attempted as an improvement in local hemostasis and miR-15b/VEGF-α reconstitution is desirable in the setting of a bleeding disorder like hemophilia A. Interestingly, miR-15b has potential binding targets in VEGF-α and is known to downregulate expression of this proangiogenic factor [14,15,21]. These data are significant as they suggest that miR-15b either alone or in combination with h.FVIII can significantly reverse the activation of hypoxia regulators or the proangiogeneic factors in arthritic joints, a data hitherto unavailable.

**Figure 6:**
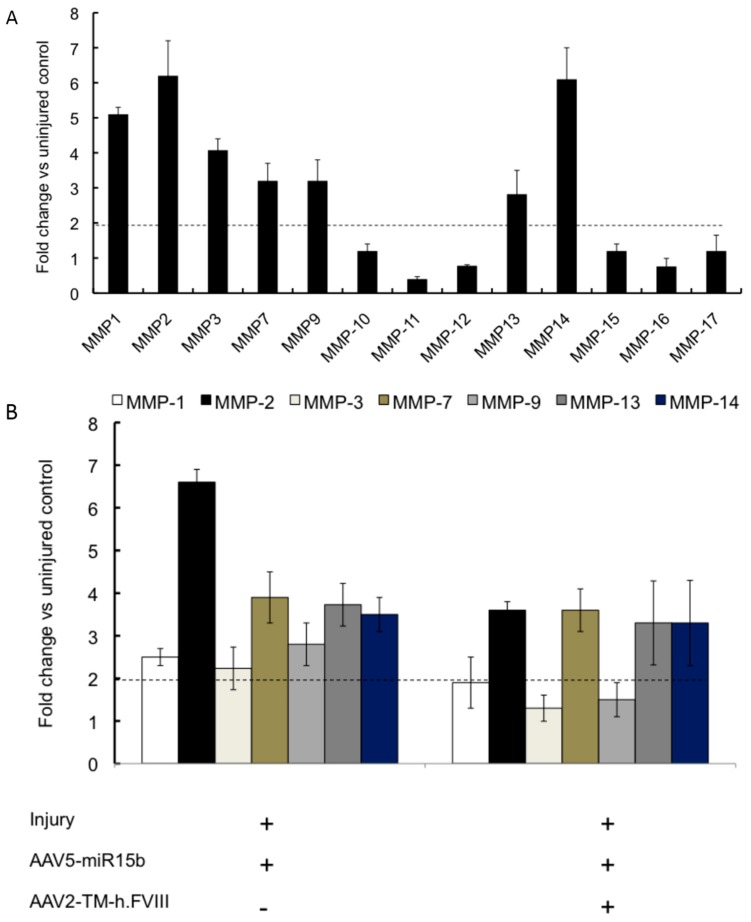
Profile of matrix metalloproteinase (MMP) in joints and the effect of AAV-miR15b/FVIII delivery in multiple injury model. (**A**) Expression levels of the various MMPs following four episodic bleeds when compared to the uninjured control; (**B**) Modulation of MMPs 1, 2, 3, 7, 9, 13, and 14, which were found to be significantly elevated in (A), by administration of AAV5-miR-15b and/or AAV2-TM-h.FVIII vectors. The horizontal dotted line denotes statistical level of significance in comparison to uninjured control joints.

We then wished to analyze the effect of miR-15b and FVIII on the group of chondrodegerative enzymes, the MMPs. As seen in Figure 6A, MMPs 1, 2, 3, 7, 9, 13 and 14 were significantly upregulated in chronic hemarthrosis model (four bleeds) compared to the uninjured control (2.8–7.3-fold). Some of these, such as MMP 3 and 9, have been previously implicated in arthritis models [22,23]. Interestingly, the combination of miR-15b and FVIII also considerably reduced the activation of MMPs 1, 3 and 9 in the injured joints (Figure 6B). For example, MMP1 levels were significantly reduced in the knee joints (~60% *vs.* 48%) upon FVIII/miR-15b gene transfer than miR-15b alone.

Taken together, our data demonstrate that the targeted overexpression of miR-15b and systemic administration of FVIII is able to significantly attenuate critical molecular mediators of joint damage.

## 3. Discussion

To the best of our knowledge, the role of miRs in the development of hemophilic arthropathy has not been studied in detail. Our study demonstrates that miR-15b is consistently downregulated in both acute and chronic hemarthrosis model of hemophilia A. Furthermore, overexpression of miR-15b in combination with FVIII significantly impacts multiple factors involved in blood induced joint damage.

The human genome contains more than 700 miRs and each miR can repress hundreds of genes, regulating almost every cellular process [24,25]. Conversely, in pathological states miRs are often dysregulated. Recent small RNA profiling studies have revealed the differential expression of miRs in comparison to corresponding healthy tissue in psoriasis and atopic eczema [26], rheumatoid arthritis [27], osteoarthritis [28], primary biliary cirrhosis [29], systemic lupus erythematosus [30] and ulcerative colitis [31]. These studies suggest that altered expression of miRs may contribute to the pathogenesis of chronic inflammatory and autoimmune diseases. Similarly, in case of hemophilia, we have now identified that one such miR-15b, is consistently repressed during joint bleeds. miR-15b, has been known to alter the expression of genes contributing to angiogenesis, hypoxia and apoptosis. For example, miR-15b regulates angiogenesis in hypoxic conditions by modulating VEGF-α levels in nasopharyngeal carcinoma cells [14,21]. miR-15b is also downregulated in hypoxic cells [14,32]. It is known to play a role in apoptosis by binding and targeting BCL2 transcripts [11,12,13]. In our previous study, we observed a progressive increase (3.3- to 4.6-fold) in hypoxic (HIF-1α/HIF-2α) and angiogeneic factors (VEGF-α) from single to multiple bleeds (1 h to 90 day after injury) [7]. Interestingly, we have now demonstrated a concomitant decrease in miR-15b levels with a maximal repression of ~4-fold after four episodic bleeds (Day 60 after first injury). Taken together, we speculate that miR-15b is linked to the three major events that lead to joint damage namely, angiogenesis, hypoxia and apoptosis.

Intra-articular delivery of coagulation factor (F) IX as a protein or gene replacement strategy is known to be protective against synovitis in hemophilia B mice [17]. Similarly, intra-articular delivery of AAV5 vector expressing TNFα antagonist has shown to significantly improve outcome in patients with rheumatoid arthritis [33]. Thus delivery of AAV based transgene into the target joints, has been consistently therapeutic by maintaining high and locally concentrated levels of the transgene. Of the different AAV serotypes available for intra-articular gene delivery, AAV5 is known to direct higher expression of the transgene [18,19,20]. In addition, their ability to remain localized to the joints is well established in contrast to other serotypes such as AAV8, which diffuse to the liver even when administered intra-articularly [17]. In concurrence to these observations, we have shown that AAV5 mediated delivery of miR-15b into the joints in combination with circulating FVIII, significantly represses the local concentrations of VEGF-α, HIF-2α and the chondrodegenetive MMPs 3, 9 in the target joints. While the basis for this remains to be elucidated, available evidence suggests that MMP3 is a direct target for miR-15b in glioma cells [21,34]. miR-15b is also shown to mediate the apoptotic effects of the anti-cancer drug dihydroartemisinin in gastric cancer cells by downregulation of MMP9 [35]. We thus reason that overexpression of miR-15b in the joints contribute to attenuation of these factors by a similar mechanism.

It is also important to recognize that we chose only a single miR-15b target for detailed investigation in this study. While the role of miRs (miR-214, miR-126) is known to contribute to endothelial diseases [36], the multifactorial etiology of hemophilic arthropathy is likely to involve other miRs) that further regulate the target molecules involved in arthropathy (VEGF-α, HIF-2α and MMP 3 and MMP 9). For example miR-15b is part of a large family including miR-15-1, 16-1, 16-2, 195 and 497 all with similar seed sequences. These yet unidentified miRs may act synergistically in a network to contribute to the progression of this disease from the onset of bleeding to development of joint disease. It is also likely that miR-15b modulates other competing target genes such as those reported for other microRNAs like miR-608 [37]. Further detailed studies including comprehensive small RNA profiling in specific synovial cell lines or with primary samples are needed to confirm these possibilities. This is likely to generate a comprehensive data matrix of the role of specific miRs and target genes in hemophilic joint disease. Nonetheless, our study provides a basis for the first time to: (1) comprehensively screen miRs from the onset of bleeding to the development of arthropathy so as to define miR based biomarkers; (2) understand mechanisms behind dysregulation of crucial molecular mediators and their pathogenic contribution to arthritis in a stage-specific manner; and (3) to design miR based therapeutics for minimizing joint disease in hemophilia.

## 4. Experimental Section

### 4.1. Hemarthrosis Model

The study was approved by Institutional review board at the Christian Medical College (CMC), Vellore, India. All experimental protocols were approved by CMC, Vellore animal care and ethics committee. The experiments were carried out in accordance with these approved guidelines. Hemophilia A mice ^(F8*−/−* E16 FVIII B6; 129s4-f8 tm1kaz)^ were obtained from Jackson’s Laboratory (Bar Harbor, ME, USA). The hemarthrosis model was generated as described previously [7]. Briefly, groups of eight to sixteen weeks old hemophilia A (*F8^−/−^*) were anesthetized. To induce hemorrhage, right knee joint capsule of each mouse was punctured with a 30 g needle below the patella. The left knee joint served as the control. Injury was repeated every 14 days to recapitulate the effects of single (Day 1 and Day 14) and multiple (Days 30/60/75/90) bleedings. Subsequently, mice were euthanized at the above-mentioned time points for various molecular and protein studies.

### 4.2. Vector Construction

The miR-15b-5p gene (NCBI reference: NC_000069.6) was amplified from mouse genomic DNA using primers, 5′-ACTACCGGTGGAGATGATTACGAAGTC-3′ and 5′-ATTAAGCTTGCTCGTAATGCAGTAGATGGC-3′ containing *AgeI* and *HindIII* restriction sites (underlined sequence), respectively. The amplified product (~500 bp) was directionally cloned within the inverted terminal repeats (ITR) of an adeno-associated virus (AAV) vector containing the chicken β actin promoter to obtain a pAAV-CB-miR-15b plasmid.

The plasmid, pAAV-HLP-codop-h.FVIII-N6 was a kind gift from Amit Nathwani (University College, London, UK) [38]. An AAV2 serotype triple mutant capsid vector (AAV2-TM) mutated at residues S489A, T251A, K532R was generated as described previously [39].

### 4.3. Generation of AAV Vectors

Self-complementary AAV vectors of serotype 5 containing miR-15b (AAV5-miR-15b) or luciferase (scAAV5-Luc) and single-stranded AAV serotype 2 vectors containing human factor VIII (AAV2-TM-h.FVIII) were generated by polyethyleneimine (PEI, Sigma-Aldrich, St. Louis, MO, USA) based triple transfection of AAV-293 cells [39].

### 4.4. Validation of miR-15b Vectors

MTT (3-(4,5-dimethylthiazol-2-yl)-2,5-diphenyltetrazolium bromide; thiazolyl blue) assay Murine fibroblast cell line (NIH3T3) cells were transfected using PEI with increasing concentrations (50 ng to 1.5 µg) of the CB-miR-15b plasmid. About 48 h later, MTT assay was performed according to the manufacturer’s protocol (Sigma-Aldrich).

### 4.5. In Vitro Studies

To check the transgene expression from the miR-15b plasmid, NIH3T3 cells were transfected with ~250–500 ng of pCB-miR-15b. Subsequently, total RNA was harvested and a quantitative (q)PCR was performed as described below. Similarly, human cervical carcinoma (HeLa) cells were mock infected or infected with 5 × 10^3^ vgs/cell of either AAV5-Luc or AAV5-miR-15b vectors. After 48 h, miR-15b levels were measured by qPCR.

### 4.6. In Vivo Gene Delivery: Optimization of Intra-Articular Vector Delivery in Hemarthrosis Model

To determine the earliest time-point at which the AAV vector injected intra-articularly, express their transgene, ~5 × 10^9^ vgs of a reporter virus, AAV5-luc was administered in a 10 µL volume into the left knee joint of hemophilia A mice. Phospate buffered saline (PBS) was administered in right knee joints to serve as the control group (Day 0). Transgene expression was measured by serial bio-luminescence imaging on Days 0, 1, 3, 5, 7 and 14 after intraperitoneal administration of the substrate, d-Luciferin (BioVision Inc., Milpias, CA, USA) in an IVIS Spect-CT small animal imaging system (Perkin Elmer, Caliper Life Sciences, Hopkinton, MA, USA). We then tested if intra-articular gene expression is persistent after multiple hemarthrosis. Groups of hemophilic mice were followed up for a further two weeks until two episodic bleedings (Day 14 and Day 21) after administration of ~5 × 10^9^ vgs AAV5-luc or AAV5-miR-15b vectors. The control or injured joint tissue from these mice was pooled and processed to isolate the total RNA.

### 4.7. Systemic h.FVIII and Intra-Articular miR-15b Gene Transfer

Hemophilia A mice were mock administered or administered intra-articularly with 5 × 10^9^ vgs of AAV5-miR-15b vectors. These mice were then randomized for intravenous injection of AAV2-TM-h.FVIII vectors at a dose of 5 × 10^10^ vgs per animal. Repeated injury was carried out every 14 days until Day 60 after the first injury (or 67 days after gene transfer) to recapitulate the effects of acute and chronic hemarthrosis. The experimental schedule is shown in Supplementary Material Figure S3.

### 4.8. Quantitative PCR

Joint specific total RNA from the control or injured joint tissues were first isolated (NucleoSpin^®^ kit; Macherey-Nagel, GmbH, Duren, Germany). cDNA was synthesized from approximately 1 μg of RNA by reverse transcription (Life Technologies, Carlsbad, CA, USA). Qualitative PCR amplification of collagen type 2 A (COL2A) gene was performed to check the joint specificity of the RNA isolated. The expression of key genes, such as HIF-1α; HIF-2α; VEGF-α; MMPs 1, 2, 3, 7, 8, 9, 10, 11, 12, 13, 14, and 15; and the housekeeping gene Glyceraldehyde 3-phosphate dehydrogenase (GAPDH) were measured by qPCR according to the manufacturer’s protocol (SYBR Premix ExTaq, Clontech, Mountain View, CA, USA). The primers for murine VEGF-α, HIF-1α, HIF-2α and GAPDH have been described [7] while murine MMP genes were amplified using primers listed in Supplementary Material Table S1.

Mature miRs were reverse transcribed using the miRCURY LNA™ Universal RT microRNA PCR protocol (Exiqon, Woburn, MA, USA). The primers to amplify mature miR-15b and SNORD68 (house keeping control) were obtained from a commercial source (Exiqon). qPCR was performed with 4 μL of the reverse transcribed miR in a total volume of 20 μL according to manufacturer’s protocol (Clontech). The relative gene expression between injured and control joints were measured by the comparative threshold cycle (2^−∆∆*C*t^) method.

### 4.9. Immunoblotting

Equal volume (20 μL) of concentrated pooled serum from 4 mice was electrophoresed by SDS-PAGE as described previously [7]. The h.FVIII was detected using the factor VIII light chain antibody (H-100, SantaCruz Biotechnology, Dallas, TX, USA) while albumin (Pierce, Rockford, IL, USA) was detected as a loading control.

### 4.10. Statistical Analysis

Multiple comparisons between groups were performed with analysis of variance (ANOVA) tests using GraphPad Prism Software. *p* value <0.05 were considered as statistically significant.

## Supplementary Materials

Supplementary materials can be found at http://www.mdpi.com/1422-0067/17/4/492/s1.
