# Supplementary material for: MicroRNA-15b Modulates Molecular Mediators of Blood Induced Arthropathy in Hemophilia Mice
Source: Int J Mol Sci. 2016 Apr 8;17(4):492. doi: 10.3390/ijms17040492 (PMC4848948; doi:10.3390/ijms17040492)
Supplement: Supplementary file 1 [file ijms-17-00492-s001.pdf]

Supplementary Materials: MicroRNA-15b Modulates Molecular Mediators of Blood Induced Arthropathy in Hemophilia Mice

Dwaipayan Sen and Giridhara R. Jayandharan

Table S1. Primers used for real time PCR amplification of MMP 1–17 target genes.

Primer	Sequence: 5′ to 3′
MMP-1-sense	AGTCTTTGAGGAGGAAGGCGATA
MMP-1-anti-sense	CAAACCTAGGCCTGGCAGAA
MMP-2-sense	CAGGGAATGAGTACTGGGTCTATT
MMP-2-anti-sense	ACTCCAGTTAAAGGCAGCATCTAC
MMP3-sense	AGGAAATCCCACATCACCTACAG
MMP3-anti-sense	TTTTCAATGGCAGAATCCACACT
MMP-7-sense	TGAATTTGGCCACTCTCTGGGTCT
MMP-7-anti-sense	TCTGAATGCCTGCAATGTCGTCCT
MMP-8-sense	AATCCTTGCCCATGCCTTTCAACC
MMP-8-anti-sense	CCAAATTCATGAGCAGCCACGAGA
MMP-9-sense	AATCTCTTCTAGAGACTGGGAAGGAG
MMP-9-anti-sense	AGCTGATTGACTAAAGTAGCTGGA
MMP-10-sense	GACCCCAGACAAATGTGATCCT
MMP-10-anti-sense	TTCAGGCTCGGGATTCCA
MMP-11-sense	CAGCGTGTTCCTCTTTCCAT
MMP-11-anti-sense	AAGAGCTCTCCTCGGATGGT
MMP-12-sense	CCCAGAGGTCAAGATGGATG
MMP-12-anti-sense	AAGTCTCCGTGAGCTCCAAA
MMP-13-sense	TAGATGGGAAACATCAGGGC
MMP-13-anti-sense	TGATGAAACCTGGACAAGCA
MMP14-sense	CCCTAGGCCTGGAACATTCT
MMP14-anti-sense	TTTGGGCTTATCTGGGACAG
MMP-15-sense	CCTCCCACGACAAGAACTGT
MMP-15-anti-sense	GGAAAGAAGCTGCAGAATGC
MMP-16-sense	GGAGACAGTTCCCCATTTGA
MMP-16 anti-sense	CGTTGGAATGTTCCAGTCCT
MMP-17-sense	CAGGAGGAACTGTCCAAAGC
MMP-17-anti-sense	CCCTCCAAGAAAGGTTCCTC

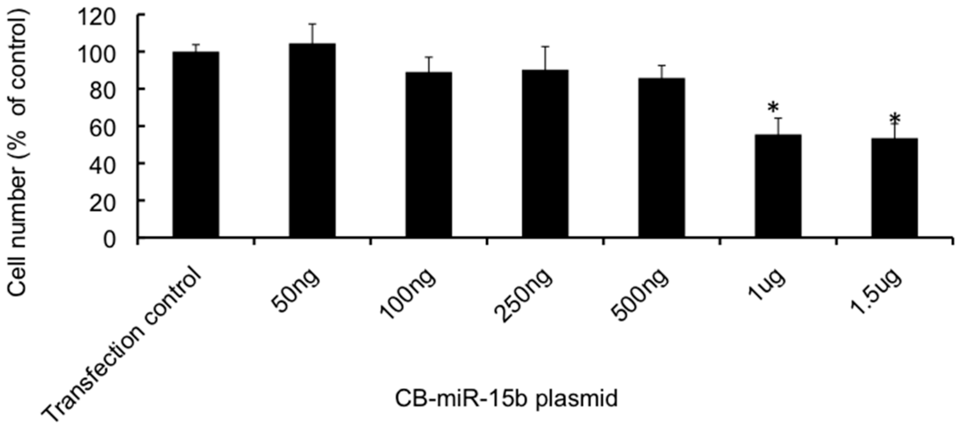

Figure S1. *In vitro* dose finding studies with CB-miR-15b plasmid vectors. NIH3T3 cells were transfected with various concentrations of CB-miR-15b plasmid. After 48 h, MTT assay was performed. Cell viability is represented as percentage of control cells that were mock transfected. $n = 6$ replicates for each condition, * $p < 0.05$.

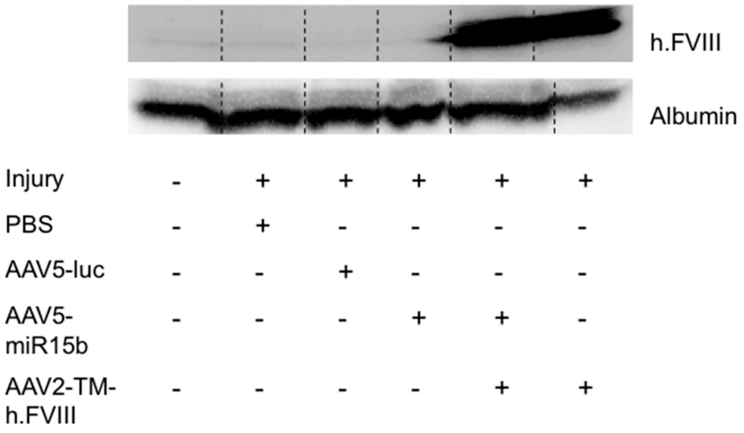

Figure S2. Immunoblotting for h.FVIII light chain. FVIII was detected in pooled serum from the different groups of haemophilia A mice. Albumin levels were concurrently determined and compared as loading controls.

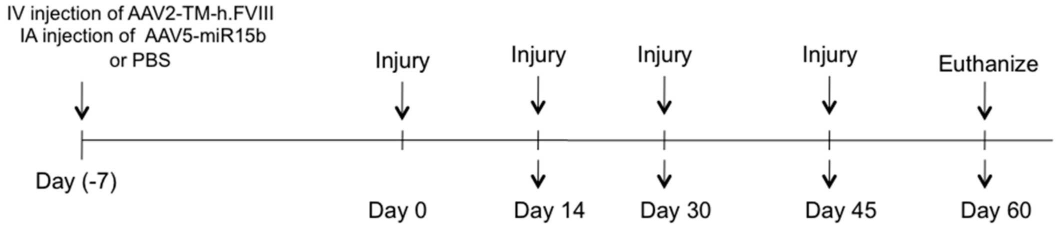

Figure S3. Schedule of gene transfer and injuries in the multiple hemarthrosis model. A week before first injury, animals received PBS or AAV vectors expressing Luc or the miR-15b Intra-articularly (left knee) at a dose of 5×10^9 vgs/knee joint. Groups of animals were also adminstered with 5×10^9 vgs/animal of AAV2-TM-h.FVIII vectors *via* the tail vein. Seven days post vector administrations, the first injury was inflicted on the left knee (day 0) using a 30g needle. Subsequent reinjuries were done at an interval of 14 days followed by euthanization at day 60 for further histological, molecular and biochemical studies. IV-Intravenous, IA-Intra-articular.
